# Single-Copy Nuclear Genes Place Haustorial Hydnoraceae within Piperales and Reveal a Cretaceous Origin of Multiple Parasitic Angiosperm Lineages

**DOI:** 10.1371/journal.pone.0079204

**Published:** 2013-11-12

**Authors:** Julia Naumann, Karsten Salomo, Joshua P. Der, Eric K. Wafula, Jay F. Bolin, Erika Maass, Lena Frenzke, Marie-Stéphanie Samain, Christoph Neinhuis, Claude W. dePamphilis, Stefan Wanke

**Affiliations:** 1 Institut für Botanik, Technische Universität Dresden, Dresden, Germany; 2 Department of Biology and Institute of Molecular Evolutionary Genetics, The Pennsylvania State University, University Park, Pennsylvania, United States of America; 3 Department of Biology, Catawba College, Salisbury, North Carolina, United States of America; 4 Department of Biological Sciences, University of Namibia, Windhoek, Namibia; 5 Instituto de Ecología, A.C., Centro Regional del Bajío, Pátzcuaro, Michoacán, Mexico; University of Ottawa, Canada

## Abstract

Extreme haustorial parasites have long captured the interest of naturalists and scientists with their greatly reduced and highly specialized morphology. Along with the reduction or loss of photosynthesis, the plastid genome often decays as photosynthetic genes are released from selective constraint. This makes it challenging to use traditional plastid genes for parasitic plant phylogenetics, and has driven the search for alternative phylogenetic and molecular evolutionary markers. Thus, evolutionary studies, such as molecular clock-based age estimates, are not yet available for all parasitic lineages. In the present study, we extracted 14 nuclear single copy genes (nSCG) from Illumina transcriptome data from one of the “strangest plants in the world”, *Hydnora visseri* (Hydnoraceae). A ∼15,000 character molecular dataset, based on all three genomic compartments, shows the utility of nSCG for reconstructing phylogenetic relationships in parasitic lineages. A relaxed molecular clock approach with the same multi-locus dataset, revealed an ancient age of ∼91 MYA for Hydnoraceae. We then estimated the stem ages of all independently originated parasitic angiosperm lineages using a published dataset, which also revealed a Cretaceous origin for Balanophoraceae, Cynomoriaceae and Apodanthaceae. With the exception of Santalales, older parasite lineages tend to be more specialized with respect to trophic level and have lower species diversity. We thus propose the “temporal specialization hypothesis” (TSH) implementing multiple independent specialization processes over time during parasitic angiosperm evolution.

## Introduction

**Figure 1:**
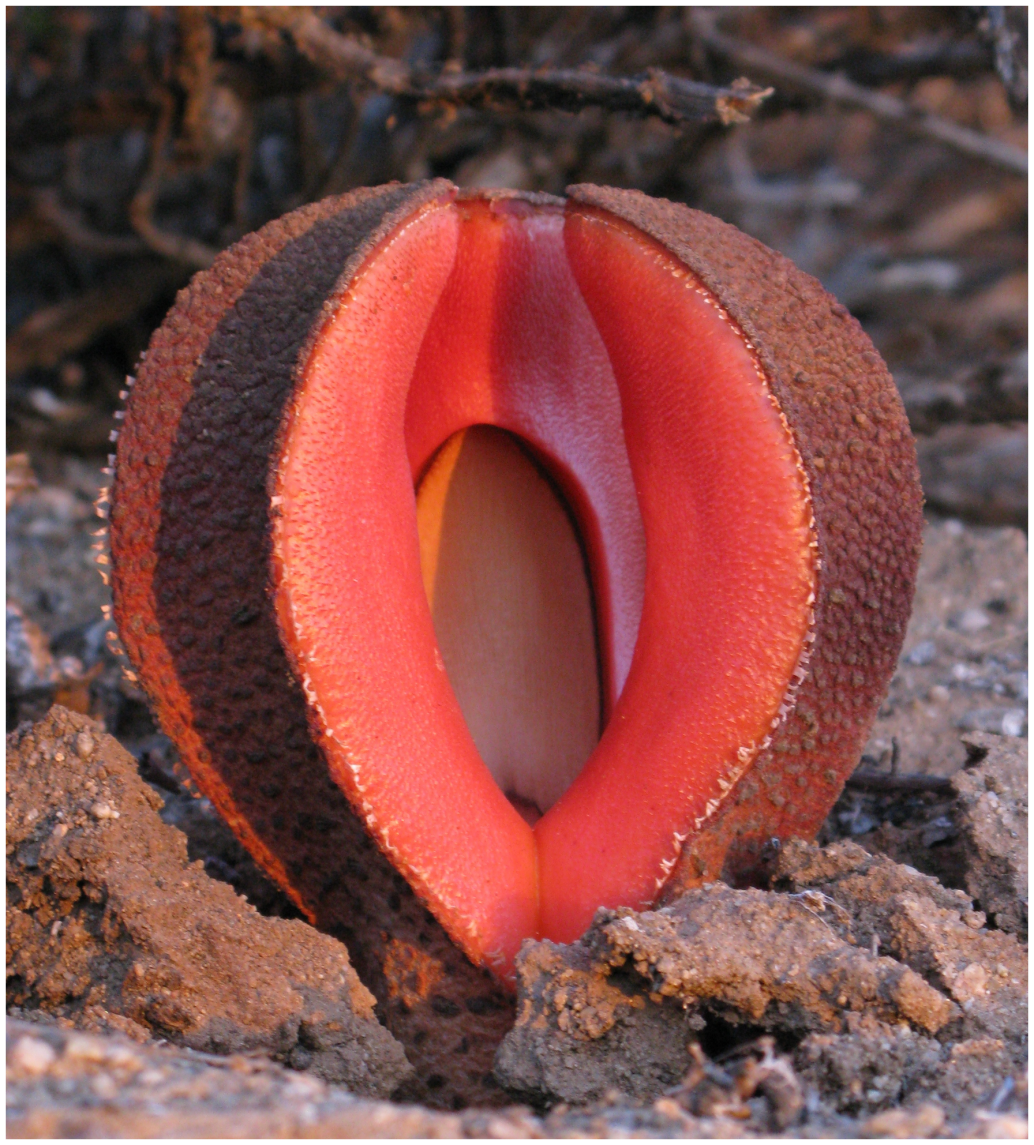
The emergent trilobed flower of *Hydnora visseri.* This photograph was taken at the type locality (Farm Namuskluft) in the Richtersveld region of southwestern Namibia. The host of *H. visseri* is *Euphorbia gummifera* at the type location (host not pictured).

Hydnoraceae have been named “the strangest plants in the world” [1] due to their weird, mushroom-like appearance with fleshy orange or whitish flowers (Figure 1) attracting dung beetles for pollination [2], the complete loss of photosynthesis, and the questionable homology of morphological structures [3] characteristic of typical plants such as root, stem, and leaves. They are also the only holoparasitic plants from among the survivors of the earliest angiosperm lineages (i.e. basal angiosperms), and Hydnoraceae include one of the few angiosperm species where flowering occurs entirely below ground (i.e. *Hydnora triceps*) [3]. This family of root-feeding parasitic plants is quite small, with about 10 species mainly distributed across the southern hemisphere of the Old World (*Hydnora*) and the New World (*Prosopanche*) [4]. Like other holoparasitic lineages, Hydnoraceae presents a challenging case for reconstructing phylogenetic relationships. This challenge stems from a highly modified (or missing) plastid genome with none of the commonly used plastid markers detected [5]–[8] (see methods). Only 10 years ago molecular markers first revealed that Hydnoraceae were relatives of black pepper (i.e. the basal angiosperm order Piperales) [6]. In addition, rate acceleration in some mitochondrial and nuclear ribosomal genes has hindered the reconstruction of phylogenetic hypotheses in parasitic plants in general [9], [10].

Until this study, only mitochondrial and nuclear ribosomal markers have been used to resolve phylogenetic relationships of Hydnoraceae [6], [10]. Here, we introduce a set of 14 highly conserved nuclear single-copy genes (nSCG) that are shared among angiosperms [11]. Nuclear single-copy genes have been used successfully to reconstruct phylogenetic relationships at species level (*Peperomia, Aristolochia*) [12], [13], family level (Brassicaceae) [11] and across angiosperms [11], [14]. These markers are a valuable option when chloroplast loci are missing or evolve too slowly to provide adequate resolution, and they provide independent phylogenetic estimates even when chloroplast loci are available. We demonstrate that these genes can complement studies based on mitochondrial and nuclear ribosomal genes, and have the potential to greatly expand the repertoire of broadly useful nuclear markers for plant phylogenetics. The nSCGs contribute substantially to fully resolving the relationships of Hydnoraceae in the Piperales and provide datasets that enable sequence-based age estimates.

In order to understand the evolution of parasitic plants, timing of their origins is needed. Although molecular clocks have been applied to datasets containing some parasitic angiosperms [15]–[19], differences in the dating approach, calibration points, and sampling strategy makes it difficult to compare ages across the range of parasite lineages. In the present study, a relaxed molecular clock was applied to the broadest phylogenetic study of parasitic plants to date, where at least eleven independent origins of haustorial parasitism in angiosperms were suggested [10]. The relative ages obtained for parasitic angiosperm lineages lead to novel insights into the diversification of the parasitic lifestyle in plants.

## Results and Discussion

### Exploring Single-copy Nuclear Genes in Hydnoraceae

The full data set of the present study consists of 19 genes (14 nSCG and 5 conventional markers (18S, *rbcL*, *atpB*, *atpA*, *matR*, Table S1, S2)) and important characteristics of the 14 nSCG are provided in Table 1. Based on the results reported by Nickrent and co-authors a decade ago [6] placing Hydnoraceae in the Piperales, we expand the taxon sampling among basal angiosperms and include all families and subfamilies in the order Piperales according to recent phylogenetic results [8], [20], [21]. The nSCG coding sequences from *Hydnora visseri*, a recently described species [22], were extracted from mRNA-seq Illumina data, while orthologous sequences were extracted from published studies, publicly available transcriptome or genome datasets, or amplified via PCR and sequenced with Sanger sequencing. The detailed sampling can be viewed in Table S1.

**Table 1:** Single copy nuclear genes used in the 19-gene-matrix. Annotations are given for the indicated *Arabidopsis* gene.

Gene homologin *A. thaliana*	Annotation	LengthCDS in*A. thaliana*	length intronwithin CDS in*A. thaliana*	alignmentlength in19-gene-matrix	number ofPICs
At2G13360	Alanine-Glyoxylate-Aminotransferase (*agt1*)	1218	284	1214	593
At3G47810	MAIGO1 (*mag1*)	573	1200	554	239
At2G32520	Carboxymethylenebutenolidase	720	909	714	396
At3G52300	ATP synthase subunit d (*atpQ*)	507	934	497	251
At5G06360	Ribosomal protein S8e family protein	783	905	781	352
At5G04600	RNA-binding family protein	669	1131	506	322
At2G21870	Male Gametophyte Defective1 (*mgp1*)	723	1455	603	352
At4G33250	Eucaryotic Initiation Factor 3 subunit K (*eiF3k*)	681	887	661	356
At4G30010	Unknown protein	273	0	251	169
At4G08230	Glycine-rich protein	342	1199	402	194
At4G31720	TBP-associated factor II 15 (*tafII15*)	405	1088	446	204
At4G37830	Cytochrome c oxidase-related protein	309	793	162	94
At5G47570	NADH dehydrogenase 1 beta subcomplex subunit 8 (*nduB8*)	378	1435	384	218
At5G23290	Prefoldin 5 (*pfd5*)	456	838	429	253

The 14 genes used here were originally selected from a set of 959 genes shared by 4 angiosperms in single copy by Duarte et al. [11]. The alignment length excludes sites of uncertain homology. Abbreviations: PICs: Parsimony informative characters. For more statistics on the 19-gene-matrix see Table S2.

**Figure 2:**
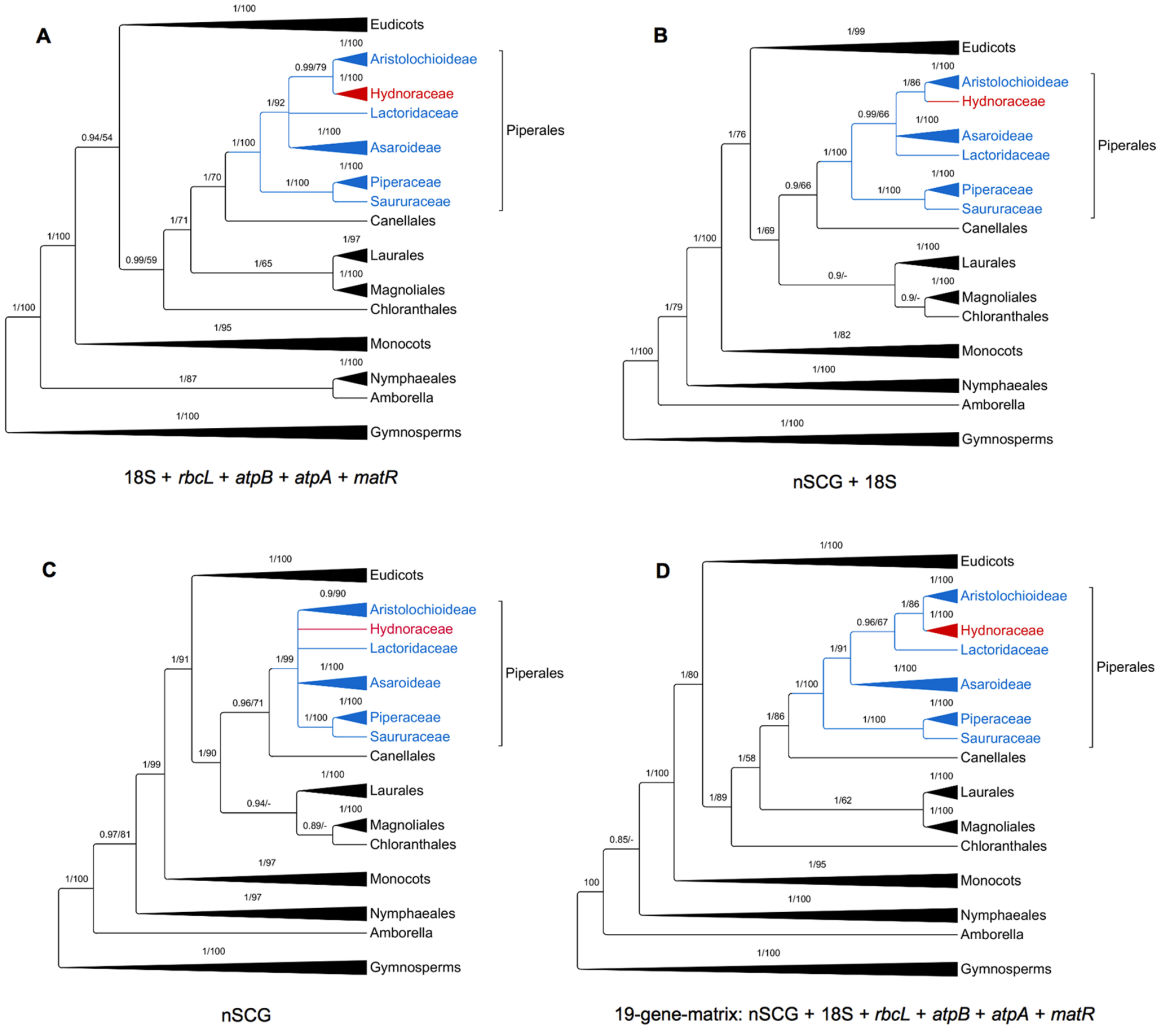
Phylogenetic origin of Hydnoraceae within photosynthetic Piperales. To compare the performance of individual marker combinations, separate analyses were run and are summarized here. The plastid *rbcL* and *atpB* genes are not available for Hydnoraceae. The phylogenetic trees are displayed at the ordinal level, but zoomed in to family level within Piperales (blue). Hydnoraceae are highlighted in red. Both Maximum Likelihood and Bayesian Inference were applied. Nodes with less than 0.85 posterior probability (PP) were collapsed, while nodes with less than 50% bootstrap support (BS) are indicated with a dash. Support values are plotted above branches (PP first, BS second). The obtained topologies are congruent at these levels, but vary in resolution within Piperales. The concatenated dataset that contains all markers (Figure 2D) provides both the best resolution and best support values. In this tree, all Piperales families are statistically supported as monophyletic (considering PP values). Nuclear ribosomal and mitochondrial markers have been calculated separately as well, but those phylogenetic hypotheses are poorly resolved (Figure S1E–F).

In order to test the applicability of nSCG for reconstructing phylogenies of lineages released from autotrophy, six partitioned datasets were constructed: a) conventional markers only, b) nSCGs and nuclear ribosomal genes only, c) nSCG only, d) all markers (19-gene-matrix), e) mitochondrial markers only and f) nuclear ribosomal marker only (Figure 2, Figure S1A–F). The latter two largely lack resolution of the angiosperm “backbone” (Figure S1A–F). Within Piperales, there are no statistically supported conflicts between the topologies. The sister relationship of Saururaceae and Piperaceae is fully supported in all trees, and this clade is sister to the Asaroideae, Aristolochioideae, Lactoridaceae and Hydnoraceae. However, the trees obtained from the nSCG plus the nrDNA marker and from the nSCG alone are not fully resolved at the family and subfamily levels. Only the combination of all conventional markers with the nSCG dataset (19-gene-matrix) fully resolves these relationships. This analysis yielded the highest support for nodes in Piperales. A grade is recovered consisting of Asaroideae followed by Lactoridaceae and a sister group relationship of Hydnoraceae and Aristolochioideae. These results highlight the valuable impact of nSCG on the dataset. About half of the data are comprised of nSCG and they contribute a significant proportion of the parsimony informative characters in the data matrix (68%, Table S2). Short branches between the ancestral node of Piperales, Asaroideae and Lactoridaceae have previously caused significant problems in reconstructing relationships within Piperales [7], [8], [20]. Besides the taxon sampling in previous studies, this pattern of short-deep and long-shallow branches is known to make marker selection difficult. It is known that these patterns often cause problems in fully resolving phylogenetic relationships within a taxonomic group (e.g. *Hydrangea s.l.*) [23], [24].

**Figure 3:**
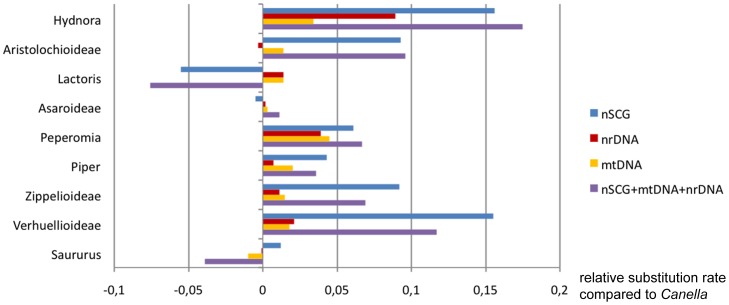
Relative substitution rates of nuclear single copy genes (nSCG) are elevated. The relative substitution rates are shown for five partitioned datasets representing major Piperales lineages. In general, nSCG contribute significantly to the overall rate of the 19-gene-matrix in the different Piperales lineages. Within Hydnoraceae, rates of nSCG regions are 2–3 fold greater than nuclear ribosomal DNA (nrDNA) or mitochondrial DNA (mtDNA). However, comparing the relative rate of Hydnoraceae with other Piperales, the nSCG regions do not exceed the rate of other photosynthetic member such as Verhuellioideae. The partition of nSCG for Piperales is reduced from 14 to the 8 most complete genes for these lineages. Rates were compared using GRate (http://bioinfweb.info/Software/GRate) for different Piperales lineages using Canellales (*Canella*) as the reference and all other sampled taxa as outgroups.

In parasitic plants, the plastid genome typically shows drastic reduction in gene content and highly accelerated rates of evolution in coding genes due to the loss of photosynthesis [25], [26], [27]. Mitochondrial and nuclear ribosomal loci in some parasitic plants have also long been known to have accelerated rates of nucleotide substitution [10], [28], [29]. A recent study investigated the substitution rates of all parasitic lineages using a comparative approach, and proposed explanations for significantly accelerated rates for these plants in all three genomes [30]. For these reasons, relative substitution rates of five partitions are compared here: nuclear, nuclear ribosomal, mitochondrial and chloroplast coding genes, as well as the combination of all markers without chloroplast regions (Figure 3). Focusing on Piperales, we use *Canella* (Canellales) as a reference and the remaining dataset as outgroup for relative rate estimation using GRate version 1.0 [31]. *Canella* is especially suitable, not only because the order Canellales is sister to Piperales, but also because Canellaceae are likely to have similar low relative rates among different regions and taxa [32]. Phylograms of the five categories calculated here show an unequal distribution of short and long branches (Figure S1C, E, F). Overall, Piperales are known for their heterogeneous rate distribution among lineages [8] (Figure 3). The highest rates can be seen in the nSCG and the combination of nSCG, mtDNA and nrDNA in most Piperales genera. A closer look at Hydnoraceae also shows an accelerated rate for 18S compared to the other taxa, but the rate of the mitochondrial markers is moderate. The rate of the nSCG is accelerated, but comparable to the rates in the nonparasitic Verhuellioideae. nSCG are gaining traction for reconstructing relationships in parasitic plants and are clearly valuable complements to existing datasets, as shown recently, for example, in a detailed analysis of a conserved phytochrome gene in Orobanchaceae [33]. In conclusion, all markers have limitations, and we propose that phylogeneticists consider a variety of markers from different organelles, including multiple nSCG, to avoid artifacts caused by rate heterogeneity or other idiosyncratic biases possessed by a single marker or genomic compartment.

**Figure 4:**
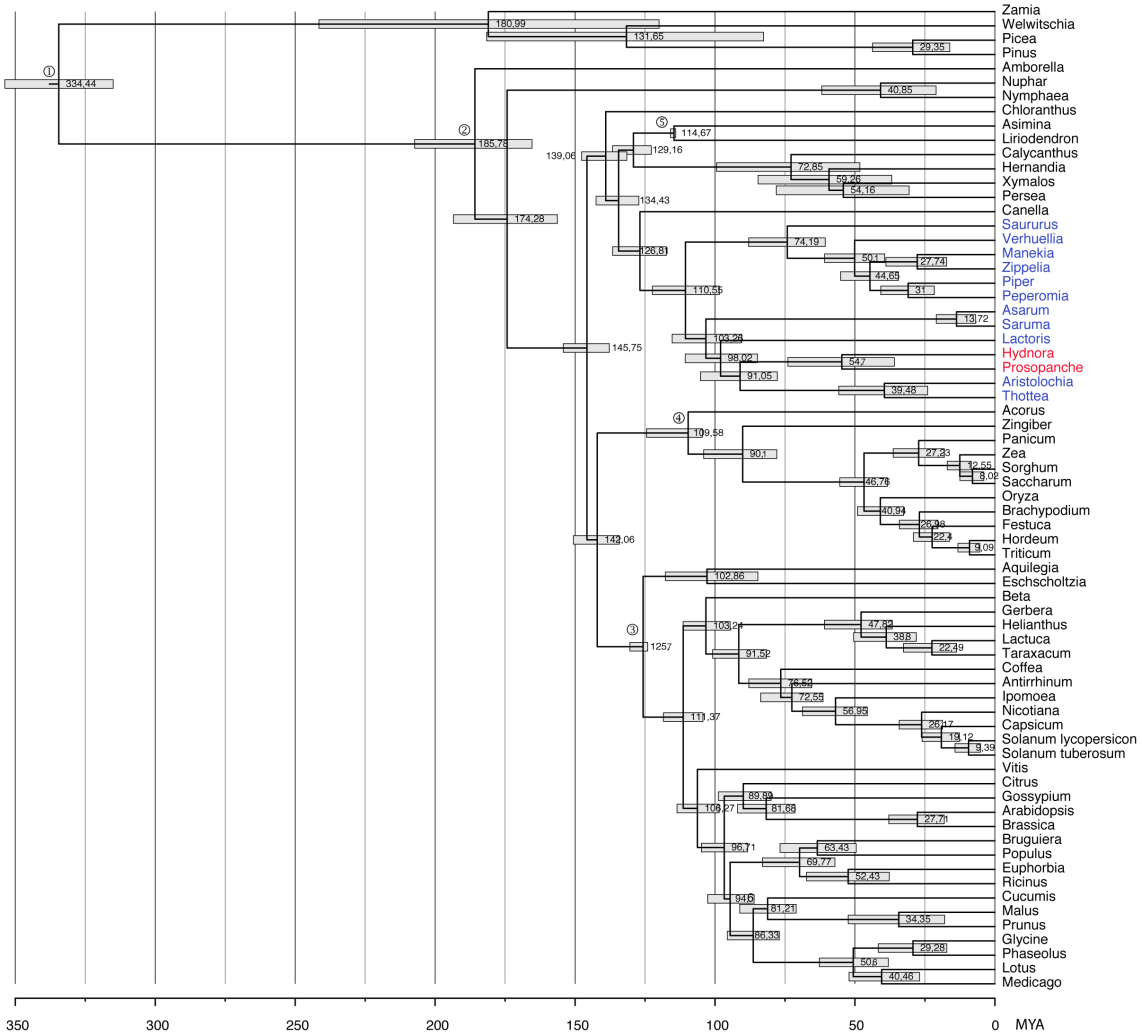
The holoparasitic Hydnoraceae originated in the Late Cretaceous. A chronogram of the 19-gene-matrix applying a relaxed molecular clock using BEAST shows Hydnoraceae (red) originating in the Late Cretaceous (91 MYA) with a crown age of 55 MYA. The photosynthetic members of Piperales are highlighted in blue. The age, estimated with BEAST [66], is mapped on the right of the respective node in MYA and the highest posterior density (HPD) interval is indicated by a grey bar. The same calibration points and topological constraints have been applied to this dataset as well as to the Barkman et al. [10] dataset to ensure comparability (for the latter see Table 2, Figure S3, and for details the methods).

The ∼15,000 bp multi-locus matrix was also used for the calculation of age estimates, yielding a stem age of ∼91 MYA (78–105 MYA, 95% Highest Posterior Density (HPD)) and a crown group age of ∼55 MYA (36–74 MYA, 95% HPD) for Hydnoraceae (Figure 4). The exclusion of the two chloroplast encoded genes from the total 19 gene matrix did not alter the age estimates for any major node within Piperales (Figure S2) These dates are indirectly confirmed by the oldest known Lactoridaceae pollen fossil (91.2 MYA), [34], [35], which was not used as a calibration point; the estimate is confirmed as only slightly younger than our age estimate for Lactoridaceae (∼98 MYA) (85–111 MYA, 95% HPD)). The surprisingly old Cretaceous origin of Hydnoraceae raises the question: What are the ages of the other haustorial parasitic angiosperms relative to Hydnoraceae and relative to each other? Transcriptomes are not yet available to extract nSCG from multiple species of all parasitic angiosperm lineages, so these results are an indication of what could be learned from comprehensive sampling of other parasitic lineages.

### The Temporal Origin of Parasitism across Angiosperms

We applied a relaxed molecular clock approach to an expanded mitochondrial dataset (*atpA*, *matR* and *coxI*; 4,500 characters) based on the Barkman et al [10] analysis. We are aware that the current taxon sampling of this dataset is insufficient for the estimations of parasitic crown group ages (CGA(s)), but maximum stem group ages (SGA(s)) do shed light, for the first time comprehensively, on the timing of the origin of parasitic angiosperm lineages. These maximum ages are valuable as secondary calibration points in future studies in the absence of a fossil record or as additional evidence in situations where the fossil record is sparse. However, based on the sampling of Hydnoraceae, and Lennoideae, minimum CGAs (58, 41 MYA respectively) are provided for these families as well as their SGAs (Table 2).

**Table 2:** Relaxed molecular clock stem group age (SGA) and crown group age (CGA) estimates for angiosperm parasitic lineages, based on the four mitochondrial marker dataset [10].

Order	Family	Species included	age in MYA (95% HPD)
Santalales	Balanophoraceae	*Ombrophytum subterraneum*	SGA: 109.75 (98.70–119.51)
	Loranthaceae	*Dendrophthoe pentandra*	
	Schoepfiaceae	*Schoepfia* sp.	
Piperales	Hydnoraceae	*Hydnora africana*	SGA: 101.38 (76.53–124.43)
		*Prosopanche americana*	CGA: 58.19 (29.54–86.89)
Ericales	Mitrastemonaceae	*Mitrastema yamamotoi*	SGA: 78.36 (55.84–98.39)
Laurales	Lauraceae	*Cassytha filiformis*	SGA: 77.33 (32.96–118.38)
Cucurbitales	Apodanthaceae	*Pilostyles thurberi*	SGA: 75.13 (58.65–91.93)
Malvales	Cytinaceae	*Cytinus ruber*	SGA: 72.11 (51.89–92.54)
“Boraginales”	Boraginaceae	*Pholisma arenarium*	SGA: 67.87 (46.39–88.41)
		*Lennoa madreporoides*	CGA: 40.86 (16.23–65.72)
Malpighiales	Rafflesiaceae	*Rafflesia pricei*	SGA: 65.29 (45.88–84.03)
		*Rhizanthes lowii*	
Zygophyllales	Krameriaceae	*Krameria lanceolata*	SGA: 61.85 (29.35–93.40)
Solanales	Convolvulaceae	*Cuscuta japonica*	SGA: 34.61 (13.09–57.04)
Lamiales	Orobanchaceae	*Epifagus virginiana*	SGA: 31.54 (12.77–51.67)

For all age estimates, we used a relaxed molecular clock approach [69]. The corresponding chronogram can be seen in Figure S3. To make the calculations comparable, topological constraints were implemented according to APGIII [71] and the same calibration points were applied.

Regarding Hydnoraceae, the CGA obtained from the 19-gene-matrix (55 MYA) (36–74 MYA, 95% HPD), Figure 4) is very similar to the date obtained from the Barkman et al. [10] dataset (58 MYA) (30–87 MYA, 95% HPD), Table 2, Figure S3), thus supporting the age estimates provided here, even though the taxon sampling is not comprehensive for all flowering plant lineages. Similarly, the Hydnoraceae SGA calculated from the mitochondrial dataset via the split from Piperaceae and Saururaceae, is placed at 101 MYA (77–124 MYA, 95% HPD). For the same split, an age of 111 MYA (99–122 MYA, 95% HPD) is calculated in the 19-gene-matrix. Due to the denser sampling of the Piperales, and in particular the presence of Aristolochioideae taxa that are more closely related to Hydnoraceae compared to Piperaceae and Saururaceae, the 19-gene-matrix provides a much more accurate SGA estimation of 91 MYA (78–105 MYA, 95% HPD) for Hydnoraceae.

Based on the Barkman et al. [10] dataset, Balanophoraceae plus Santalales appear to be the earliest diverging parasitic lineage (∼109 MYA) (99–119.5 MYA, 95% HPD), followed by Hydnoraceae (∼101 MYA) (77–124 MYA, 95% HPD) and Cynomoriaceae (∼100 MYA) (76–117 MYA, 95% HPD) (Table 2, Figure S3). Unfortunately, because the basal non-parasitic lineages of Santalales were not sampled, we are unable to determine if parasitism evolved independently in Balanophoraceae and Santalales [36]. In our study, Balanophoraceae and Santalales form a monophyletic group and will thus be treated together here. On the other end of the spectrum, parasitism evolved most recently in Orobanchaceae (∼32 MYA) (13–52 MYA, 95% HPD), preceded shortly by *Cuscuta* (Convolvulaceae, ∼35 MYA) (13–57 MYA, 95% HPD), Table 2, Figure S3).

Few studies have attempted age estimates for particular parasitic lineages. Bremer et al. [14], with the dating of asterids as their focal point, revealed an Orobanchaceae stem group age of 64 MYA, whereas the same node was calculated at 40–50 MYA by Wolfe et al. [16], both older than our age estimation (∼32 MYA) (13–52 MYA, 95% HPD), Table 2, Figure S3). Given that a Cretaceous fossil record is not known for Lamiales and only few extant families of Lamiales have a fossil record starting in the Eocene and Oligocene [37], and the only Orobanchaceae fossil currently known is Pliocene [38], the age estimate provided here is in line with the absence of an older fossil record.

Wang et al. [17], focusing on rosids, provided mean stem group age estimates for the origin of Krameriaceae between 89 and 55 MYA (46–102 MYA, 95% HPD), which is consistent with the age estimate in the present study (∼62 MYA) (29–93 MYA, 95% HPD), Table 2, Figure S3).

For the split of Rafflesiaceae and Euphorbiaceae, an age of 65 MYA (46–84 MYA, 95% HPD) is obtained here (Table 2, Figure S3). The Malpighiales crown group age is here estimated at 77 MYA (60–94 MYA, 95% HPD), and its stem group age at 88 MYA (72–103 MYA, 95% HPD). Other studies are in complete agreement; the Malpighiales crown group age (77 MYA) and stem group age (88 MYA) found in Wikström et al. [39] were identical to our estimates. However, an older stem group age estimate was provided by Bendiksby et al. [18] for Rafflesiaceae (95 MYA) (83–109 MYA, 95% HPD). Also, the split of *Rafflesia* and *Rhizanthes* is calculated at 37 MYA (18–56 MYA; 95% HPD) for our dataset and thus half as old as in Bendiksby et al. [18] (73 MYA). These differences may be due to the single secondary constraint for the crown group of Malpighiales in Bendiksby et al. [18]. Their calibration point is based on the range of mean age estimates of molecular dating studies (ranging between 77 and 115 MYA) [39], [40], [41], using the estimate of Wikström et al. [39] as a minimum offset. Furthermore, Bendiksby et al. [18] applied this age constraint to a very limited sampling of Malpighiales (two species) that may not represent the crown group age very well. Additionally, that study did not include any non-Malpighiales outgroups. As a consequence, they may have overestimated internal node ages in their study.

Vidall-Russel et al. [19] published a molecular dating study of the Santalales where the order was densely sampled but did not include Balanophoraceae. Their study estimated an age of 82 MYA for the split of Loranthaceae from a clade including Schoepfiaceae, corresponding to a node in the present study estimated at 67 MYA (36–95 MYA; 95% HPD, Table 2, Figure S3). The higher age of Vidall-Russel et al. [19] might be due to their root age constraint of 114 MYA. They assumed the split of *Saxifraga* and Santalales was equal to the Santalales crown group age, likely providing older estimates for some of the nodes.

For Apodanthaceae, Balanophoraceae, *Cassytha*, *Cuscuta*, Cynomoriaceae, Cytinaceae, Hydnoraceae, Lennoideae, and Mitrastemonaceae there are no previous age estimates available. Thus, the ages provided here represent a first glimpse into the temporal origins of haustorial parasitism across angiosperms. It is worth noting that the phylogenetic placement of Cynomoriaceae has been variously reported in the literature and that our reconstruction is different from Barkman et al. [10]. The mitochondrial dataset used here suggest Cynomoriaceae is related to Saxifragales, consistent with nuclear ribosomal and mitochondrial markers used previously [36], [42]. In contrast, chloroplast markers have placed Cynomoriaceae in Rosales [43], [44].

The age estimations of all parasitic angiosperm lineages are provided in Table 2 and Figure S3. Multiple independent origins of haustorial parasitism, and the persistence of many parasitic plants over potentially surprisingly long periods since possibly the late Cretaceous and early Paleogene, provide evidence of the success of this distinctive life strategy, and raise questions about the evolutionary fate of parasitic plant lineages. Having all parasitic lineages in a single data set, analyzed under the same conditions, allows us to detect general evolutionary patterns and make comparative observations.

### Evolutionary Trends in Parasitic Angiosperms

**Figure 5:**
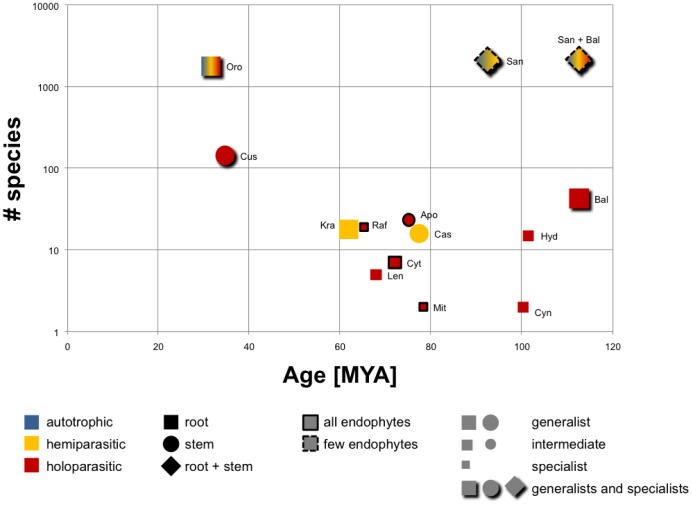
The “temporal specialization hypothesis” (TSH) postulates increasing specialization during the evolution of parasitism in plants. Relationship of stem age, species number, host range, trophic type and host attachment site of the parasitic lineages is shown. The estimated age of each parasite lineage is plotted relative to lineage size (the species numbers are taken from the review by Westwood et al. [51]). The color of the symbol represents the trophic type for the indicated lineage (blue: autotrophic; yellow: hemiparasitic; red: holoparasitic), the shape indicates the mode of attachment (square: root parasite; round: stem parasite; rhomb: stem and root parasite; rimmed: endophytic) and the size represents host range (large: generalist on more than five families; medium: intermediate host range of two to five families; small: specialist on only one host family; shaded: all types of host ranges). Santalales and Balanophoraceae are plotted separately and together since phylogenetic analyses to date are inconclusive about the origin of Balanophoraceae within Santalales [36]. As the host range is difficult to capture, we chose three categories. A lineage is categorized by the typical host range and exceptions may exist. For Hydnoraceae hosts typically occur in just two families (Fabaceae, Euphorbiaceae), however, *Prosopanche bonacinae* has a broad host spectrum of numerous families. Abbreviations: Apo: Apodanthaceae; Bal: Balanophoraceae; Cas: *Cassytha*; Cus: *Cuscuta*; Cyn: Cynomoriaceae; Cyt: Cytinaceae; Hyd: Hydnoraceae; Kra: Krameriaceae; Len: Lennoaceae (Boraginaceae sf. Lennooideae); Mit: Mitrastemonaceae; Oro: Orobanchaceae; Raf: Rafflesiaceae; San: Santalales.

Parasitic plants can vary in their degree of host-dependence: hemiparasites are photosynthetic and some do not require a host to complete their life cycle, whereas holoparasites rely completely on their host for both water and nutrients [45]. The timing of the origin of parasitism with respect to the trophic mode (holo- or hemiparasitic), host connection (stem-, root- and endoparasite) and host range (generalist versus specialist) reveals important insights to evolutionary strategies in parasitic angiosperms (Figure 5).

The trend of specialization over time is a common pattern in parasites, and has been studied intensively in insects and other organisms [46]. We see a trend toward parasitic lineages with fewer species through evolutionary time (Figure 5). However, as far as parasitic plant lineages that have been studied in detail, a recent radiation can be observed in Rafflesiaceae [18]. In general, the number of species in a parasitic lineage decreases over time as some host-parasite-interactions are reinforced and maintained over tens of millions of years, while other more specialized parasites may have gone extinct due to changes in the host landscape.

The persistence of species-poor lineages has been studied for animals in general, with evidence suggesting that species-poor lineages might actually be more common than expected under a neutral model with equal rates of lineage birth and death [47]. Two scenarios could explain this pattern in parasitic plants, where species-poor lineages are also common: (1) The extant parasitic plants are just a small fraction of many parasitic lineages that arose throughout the diversification of angiosperms, but most of them became extinct without ever radiating to become large lineages, or (2) the majority of parasitic lineages that emerged and radiated eventually then decreased to small persistent lineages of specialized taxa. We postulate a possible explanation (the “temporal specialization hypothesis, TSH”) where the evolution of parasitic plants involves a process of increasing specialization over time that is selectively advantageous due to increased efficiency of successful parasitism on particular host plants. In an early stage of parasite evolution, a parasite lineage may establish relationships with a wide range of hosts. Over time, the host-range narrows to one or a few compatible host families as the parasite gains specialized genetic information to overcome evolving host defenses, and loses genetic information required for successful parasitism on rarely encountered host species. Once a parasitic lineage is specialized, it might then persist over a long evolutionary time frame. It has been thoroughly discussed and reviewed by Colles et al. [48] that specialization does not necessarily increase the extinction risk. From our data, it is not clear to what degree specialization might ultimately be an evolutionary “dead end”, leading to extinction when a parasite-host interaction gets altered. In general, parasitic plant lineages are less species rich than their non-parasitic sister groups [49]. The overrepresentation of specialists among ancient parasitic plant lineages, however, does make a strict dead-end scenario less likely. In contrast to Colles et al. [48], specialized lineages, in our case holoparasitic plants, do not seem to give rise to generalists again, which would also be in accord with factors of ecological limitation pinpointed by Hardy and Cook [49] for parasitic plant diversification.

In terms of species numbers and other indicators of evolutionary diversification, Orobanchaceae (>1,800 spp.) and Santalales (>2,100 spp.) [50] are the most successful lineages of parasitic plants [51] and they have even greater structural diversity than their non-parasitic sisters [49]. Both Orobanchaceae and Santalales are geographically widespread and are the only parasitic plant lineages distributed throughout temperate regions. Orobanchaceae represent a very young lineage in which frequent specialization [52] has not yet resulted in a reduction in the family-wide species number. Orobanchaceae are root parasites and all trophic modes from non-parasitic, facultative, and obligate hemiparasitic to holoparasitic are represented in the family [51]. Host range also varies considerably in the family, with broad generalists that parasitize hosts from many dicot and monocot families (e.g., *Triphysaria*) [53] to extreme specialists that have only a single host species (e.g., *Epifagus virginiana* on *Fagus grandifolia*). Santalales include both non-parasitic and root and stem hemiparasites, with a few lineages trending toward holoparasitism and the endoparasitic habit (e.g. *Arceuthobium*) [54], [55]. Like Orobanchaceae, Santalales include host generalists and specialists as well as different trophic modes. Furthermore, even without Balanophoraceae, Santalales encompass greater morphological diversity than Orobanchaceae; the order includes both woody and herbaceous members and different types of parasitism (root, stem and endophytic parasites). Santalales has retained a generalist trophic mode through the diversification of most of the major lineages within the order and has remained a speciose group. This seemingly contradicts the TSH hypothesis, however, on closer examination of the order, several lineages are observed that have undergone a high degree of host specialization and represent ancient, species-poor lineages. For example, Misodendraceae (8 species) [56] are specialists on *Nothofagus* and date back to ∼80 MYA [19]. Additionally, species of Amphorogynaceae (68 species) have narrow host ranges and as a family, diverged from Viscaceae ∼72 MYA [19]. *Cuscuta* (Convolvulaceae) are stem parasites, ranging from broad generalists to specialists. The number of species in *Cuscuta* is an order of magnitude smaller than Orobanchaceae and Santalales (>145 spp.), and they are only slightly older than Orobanchaceae. Their trophic mode has narrowed to holoparasitic dependence, although most species still retain a complete functionally constrained set of photosynthetic genes, implying that they probably retain a functional photosynthetic apparatus [57]. All other parasitic lineages today have much smaller species numbers (<25 spp.), and are either strictly holo- or hemiparasitic.

The three parasitic lineages of greatest inferred age in this study, Balanophoraceae, Cynomoriaceae and Hydnoraceae, share important characteristics: they are all species-poor lineages of root holoparasites (Figure 5). This suggests two possible but not exclusive interpretations: (1) Parasitic plants may need to be highly specialized on a host in order to make the full transition to holoparasitism and the loss of photosynthesis. This may also preempt the ability of these lineages to diversify, explaining why most holoparasitic lineages are species-poor. (2) Holoparasitism could simply be the most likely parasitic strategy to persist over time. However, it might also be that a stable host environment permits long-term survival. As a parasitic lineage becomes more specialized it becomes less likely to adapt to drastic ecological changes presented by shifting host availability, potentially resulting in a higher chance of extinction. These patterns can also be observed on the genetic level of parasite-host interaction. A recent study of the haustorial transcriptome in the generalist parasite *Triphysaria versicolor* (Orobanchaceae) showed that there were large and distinct yet overlapping sets of genes expressed by *Triphysaria* at the host-parasite interface, when grown on different host species [53]. This suggested that the genetic basis of generalism in the parasite did in fact lie, at least in part, in the maintenance of a diverse set of genes with specialized functions for different host plants. Such a situation would be unlikely to be maintained long-term, except under conditions where contact with a wide range of host species regularly occurs [53]. However, since *Triphysaria* is a relatively young lineage, it is possible that this lineage has not yet experienced the progressive narrowing of host preferences, and concomitant loss of functions required for many different host plants, as seen in many other Orobanchaceae [52], and most of the older parasitic plant lineages.

### Conclusions

Nuclear single copy genes are shown to contribute valuable information towards the resolution of phylogenetic relationships in haustorial parasitic angiosperms. The introduction of 14 nuclear gene markers and increased taxon sampling help to refine, with statistical confidence, the identification of Aristolochioideae as the closest nonparasitic relatives of Hydnoraceae. Now that Hydnoraceae are confidently placed within Piperales it is possible to reappraise synapomorphies and the potential origin of morphological traits such as those related to the flower and its evolution in the perianth-bearing Piperales. A relaxed molecular clock applied to the 19-gene-matrix reveals that Hydnoraceae is an ancient parasitic lineage with a stem group age over 90 MYA. A comparison of all haustorial angiosperm parasites reveals Balanophoraceae, Hydnoraceae and Cynomoriaceae as the oldest extant parasitic lineages, each having emerged in the Cretaceous. These are the first reported age estimates for many parasitic lineages. However, sampling density is known to have a significant influence on molecular dating approaches [58] and thus age estimates provided here (mostly stem group ages) should be regarded as a starting point for future studies when nSCG data will be available for a large number of parasitic and nonparasitic taxa.

## Materials and Methods

### Generation of *Hydnora visseri* Transcriptomic Data

Plant material of *H. visseri*, a recently described species [22], was collected on private property (Gondwana Cañon Preserve) (Namibian MET Permit No. 1350/2009). The tissue was snap frozen after collection and kept frozen at −80°C. The RNA of *H. visseri* was extracted individually from 6 tissues (tepal, osmophore, androecium, gynoecium, fruit and rhizome tip) using cetyltrimethylammonium bromide (CTAB). Equal amounts of the RNA of all six tissues were pooled and two libraries were prepared with the Illumina mRNA-seq protocol; one of the libraries was normalized using the Duplex-Specific thermostable nuclease enzyme (Evrogen) following the Illumina protocol for DSN normalization. The whole plant library and the whole plant normalized library were sequenced 2×84 and 2×75 respectively on an Illumina Genome Analyzer IIx. The sequence data were assembled using the Trinity RNA-seq pipeline [59]. The resulting unigenes were sorted into the PlantTribes 2.0 database (10 genome orthogroup scaffold; http://fgp.huck.psu.edu/tribedb/10_genomes) with BLASTx and assigned putative annotation terms from functionally annotated genes in the assigned orthogroup [60]. Unigene sequences corresponding to the 14 nSCG used in this study were extracted for phylogenetic analysis.

### The 19-gene-matrix

This dataset is based on the Duarte et al. [11] single copy nuclear genes. We added sequences of 11 Piperales genera (*Aristolochia*, *Asarum*, *Hydnora*, *Lactoris*, *Manekia*, *Peperomia*, *Piper*, *Saururus*, *Thottea*, *Verhuellia*, *Zippelia*) as well as sequences of six other basal angiosperms (*Asimina*, *Calycanthus*, *Canella*, *Hernandia*, *Nymphaea*, *Xymalos*) to the dataset. The RNA of *Canella*, *Manekia*, *Nymphaea*, *Thottea*, *Verhuellia*, and *Zippelia* were extracted from snap frozen tissue using CTAB and amplified via Reverse Transcriptase PCR (Promega kit, Table S3). The RNA of *Asarum*, *Asimina*, *Calycanthus*, *Hernandia*, *Peperomia*, *Piper*, *Saururus* and *Xymalos* was extracted from snap frozen tissue using Qiagen Plant RNA kit and amplified via Reverse Transcriptase PCR (Invitrogen kit). Genomic DNA of *Lactoris fernandeziana, Verhuellia, Manekia* and *Zippelia* was isolated from silica gel dried material using CTAB [57] and amplified with traditional PCR. Introns were identified and removed from these sequences based on the respective *Arabidopsis thaliana* gene models.

The 14 nSCG matrix of Duarte et al. [11] was supplemented with additional sequences for *Amborella*, *Liriodendron*, *Nuphar*, *Persea*, *Aristolochia*, and *Zamia* from Jiao et al. [61] and data from NCBI. The 18S, *rbcL* and *atpB* sequences are taken from Nickrent et al. [6] and *atpA* and *matR* were taken from Barkman et al. [10]. For sampling details see Table S1. Sequences were aligned and edited manually with PhyDE [62]. Characters that were of uncertain homology in the alignments were masked prior to phylogenetic and molecular evolutionary analyses. Sequence statistics of the full dataset and the different organellar compartments were obtained using SeqState [63] (Table S2). The entire alignment can be viewed in Dataset S1.

### Phylogenetic Reconstruction

To obtain insights into the phylogenetic relationships within Piperales, we used the 19-gene-matrix (not containing the *cox1* region from [10] due to missing Piperales sequences) for an unconstrained phylogenetic analysis. The best model for each dataset was determined with jModelTest [64] based on the Akaike information criterion (AIC). Maximum Likelihood (ML), implementing the GTR+G model as suggested by Stamatakis in the RAxML manual, was conducted with RAxML v7.2.6 [65] using the rapid Bootstrap (BS) algorithm that is combined with the search for the best scoring ML tree. 1,000 BS replicates were applied for all analyses. Bayesian inferences (BI) were performed with MrBayes v3.2.1 [66]. Four parallel Markov chains were run for at least 2 million generations and trees were saved every 100 to 1000 generations. The burn-in was individually set for each analysis between 5 and 25% after determining stationarity of each run with Tracer v1.5 [67]. Twelve runs were combined together to generate the consensus trees and posterior probabilities. The phylogenetic trees were formatted with TreeGraph2 [68].

### Molecular Dating

Relaxed molecular clock dating analyses were performed using BEAST v. 1.7.4 [69] applying the BEAGLE v. 1.0 high-performance library [70]. Two independent analyses were performed, based on the 19-gene-matrix and on the three mitochondrial marker dataset (*matR*, *atpA*, and *cox1*) [10] including all parasitic angiosperm lineages. Additional sequence data from *Berberidopsis* (the closest non-parasitic relative of Santalales; APG III) from GenBank was added. Furthermore, the RNA editing sites for the mitochondrial dataset were excluded following Barkman et al. [10]. For both analyses, the 19-gene-matrix and the mitochondrial dataset [10], starting trees were calculated in BEAST, applying topological constraints according to APG III [71] to make them comparable. For both starting trees, the same settings as in “Phylogenetic Reconstruction” were applied and subsequently made ultrametric (setting “Arbitrary Ultrametricize”) with the Mesquite package [72].

We chose five identical calibration points for both calculations as follows: (1) the seed plant root age as normal distribution with a mean of 346 MYA and a standard deviation of 12 based on Clarke et al. [73], (2) the root age of angiosperms as uniform age, ranging 251–145.5 MYA [73], (3) the eudicot crown group age based on the tricolpate pollen fossil applying a lognormal distribution with mean in real space = 1, lognormal standard deviation = 0.5 and offset = 124 MYA, (4) the monocots crown age based on *Mayoa portugallica* [74] (lognormal distribution, mean in real space = 1, lognormal SD = 0.5, offset = 104.5 MYA), and (5) the Magnoliales crown age applying the *Endressinia brasiliana* fossil [75] with a log normal distribution, a mean in real space of 1, a lognormal standard deviation = 0.5 and a minimum age offset of 114 MYA. All calibration points are plotted to the chronograms respectively in Figure 4 and Figure S3.

The uncorrelated lognormal (UCLN) model, the GTR+Г+I substitution model and the “Birth-Death-Model for incomplete sampling” were used, as implemented in BEAST [76]. For the three mitochondrial marker dataset, 250 million generations were calculated, sampling every 5000^th^ state, and discarding the first 10 million states as burn-in.

Convergence of the Markov chains was assessed using Tracer v1.5 [67]. The effective sample size (ESS) for all parameters was over 100. Consensus trees with mean branch lengths were generated with TreeAnnotator v. 1.7.4 (part of the BEAST package).

To test if ages among young (Orobanchaceae, *Cuscuta*), medium old (Krameriaceae, Rafflesiaceae Apodanthaceae, Lennoaceae, Mitrastemonaceae, Cytinaceae *Cassytha*), and old parasitic lineages (Cynomoriaceae, Hydnoraceae, Santalales incl. Balanophoraceae) are significantly different, we performed a Kruskal-Wallis One Way ANOVA on ranks using individual ages of nodes of 45001 post burn-in trees. Stem group ages of the parasitic lineages of the 45001 trees were extracted from BEAST using a python script (nodes according to Table 2). We compared the three groupings of young, medium-old, and old with respect to statistical significant differences of ages. First, the ANOVA on ranks resulted in statistically differences among groups (p<0.001). Second, an All Pairwise Multiple Comparison procedure (Dunn’s Method) testing all possible combinations of groups against each other, revealed significant differences (P<0.01) across all comparisons.

### Relative Rates Estimation

To account for relative rate heterogeneity between different regions, a relative rate test was performed with a focus on Piperales. Setting *Canella* as a reference and the remaining taxa as outgroup, relative rates of all sampled Piperales taxa were estimated using GRate 1.0 (http://bioinfweb.info/Software/GRate) [31] and PAUP* 4.0b [77] as described in the GRate manual. This analysis was applied for each of the four regions individually (nrDNA, mtDNA, cpDNA, and nSCG) as well as a combination of nrDNA, mtDNA, and nSCG. An individual starting tree, based on the topology recovered in the unconstrained combined analysis of the 19-gene-matrix, was enforced. The MLS3 (GTR+Г+I) model was applied to estimate parameters on individual starting trees and 1000 bootstrap replicates for all datasets were performed.

## Supporting Information

Figure S1 Phylogram of: A) traditional markers (18S, *rbcL*, *atpB*, *atpA*, and *matR*), B) nuclear markers (nSCG and 18S), C) nSCG only, D) 19-gene-matrix (nSCG, *rbcL*, *atpB*, *atpA*, and *matR*), E) nuclear ribosomal marker (18S) only, F) mitochondrial marker (*atpA* and *matR*) only, G) nSCG, nuclear ribosomal marker (18S), and mitochondrial marker (*atpA* and *matR*), H) plastid marker (*rbcL* and *atpB*) only, obtained from BI. Support values were mapped above branches: PP on left, BS obtained from ML on right. BS values below 50% are indicated with a dash. This figure is related to Figure 2, where a summary of this phylogenetic tree is shown.
(PDF)

Figure S2 A chronogram, based on the full dataset but excluding the two chloroplast regions, applying a relaxed molecular clock using BEAST shows Hydnoraceae (red) originating in the Late Cretaceous (91 MYA) with a crown age of 54 MYA. The photosynthetic members of Piperales are highlighted in blue. The age is mapped on the right of the respective node in MYA and the highest posterior density (HPD) interval is indicated by a grey bar. Identical calibration points and topology constraints have been applied to all datasets to ensure comparability (Table 2, see methods for details).
(TIF)

Figure S3 Chronogram of the mitochondrial marker dataset [10], related to Table 2. This figure shows the calculated ages obtained from the relaxed molecular clock analyses inferred in BEAST including 95% HPD intervals and applied age constraints. Identical calibration points and topology constraints have been applied to all datasets to ensure comparability (Table 2, see methods for details).
(TIF)

Table S1 Species names and GenBank accession numbers of all sequences in the 19-gene-matrix, related to Figure 2–4.
(XLS)

Table S2 Sequence statistics of the nuclear, plastid, and mitochondrial genes of the 19-gene-matrix obtained using SeqState [63]. Outgroup taxa have been removed for the calculations.
(XLS)

Table S3 Primer sequences designed for the present study. The region name is based on the *Arabidopsis thaliana* gene model [11]. Primers were used for both genomic DNA and cDNA amplification.
(XLS)

Dataset S1 The 19-gene-matrix.
(NEX)
