# Supplementary figures and images for: Single-Copy Nuclear Genes Place Haustorial Hydnoraceae within Piperales and Reveal a Cretaceous Origin of Multiple Parasitic Angiosperm Lineages
Source: PLoS One. 2013 Nov 12;8(11):e79204. doi: 10.1371/journal.pone.0079204 (PMC3827129; doi:10.1371/journal.pone.0079204)

A

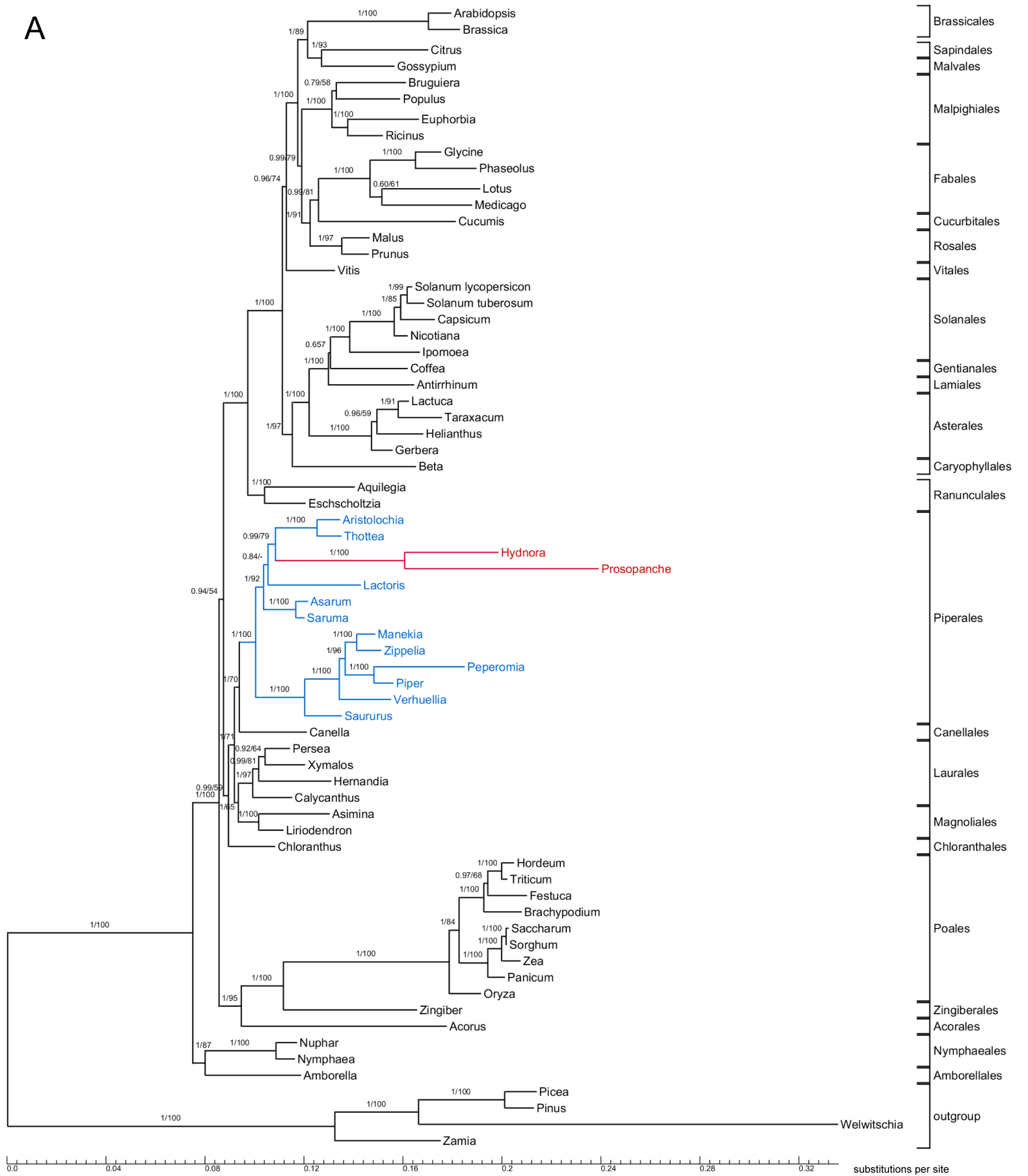

B

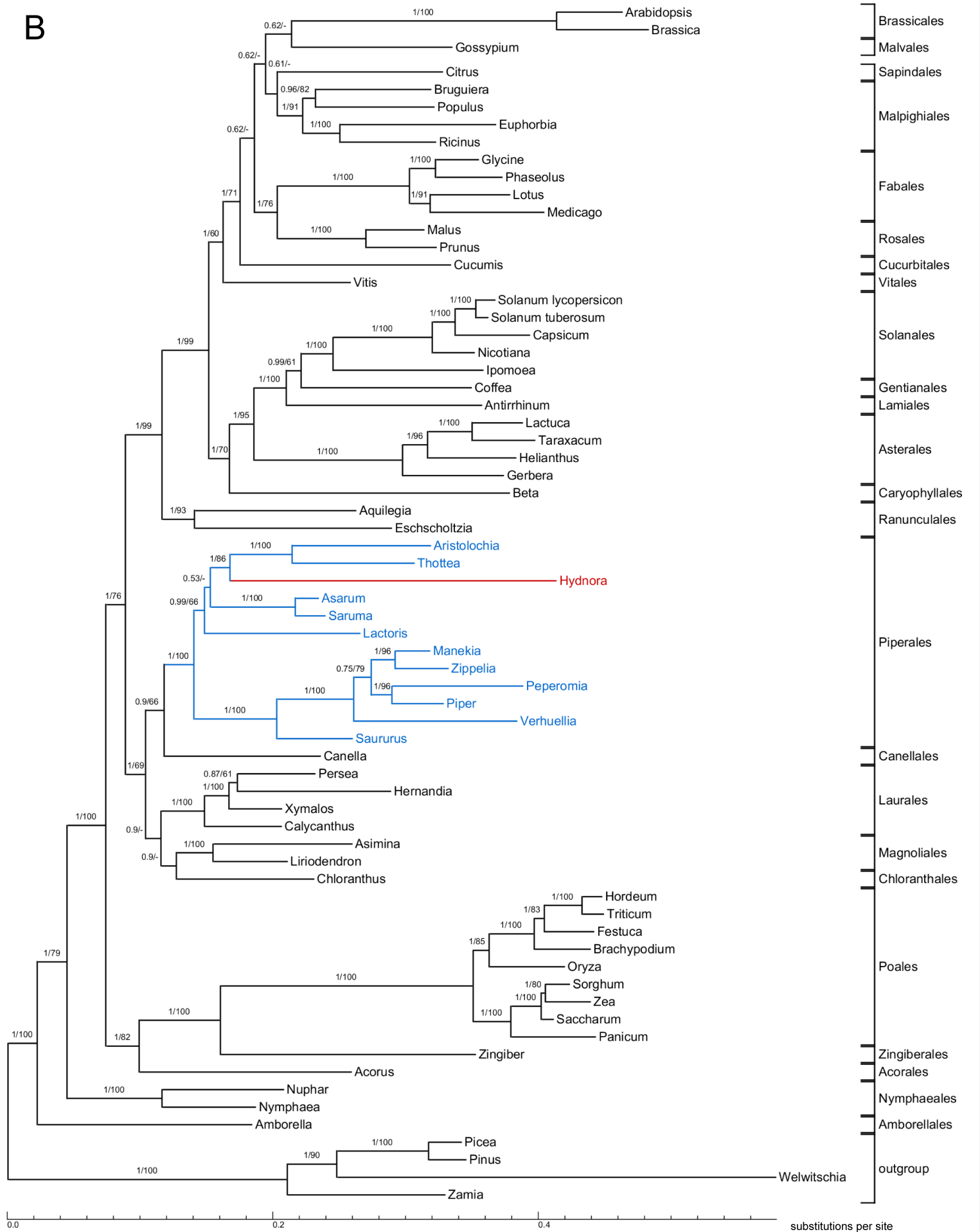

C

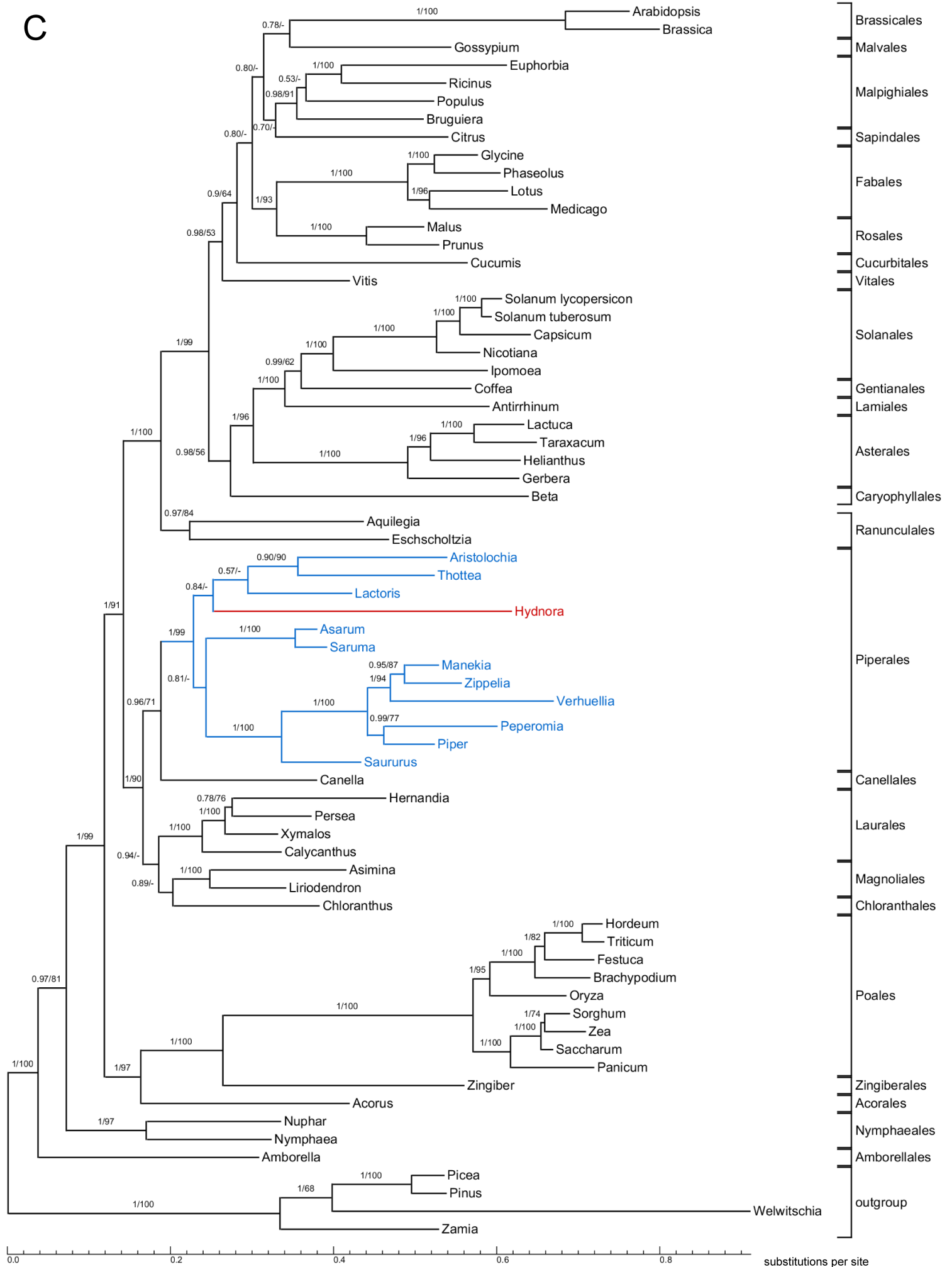

D

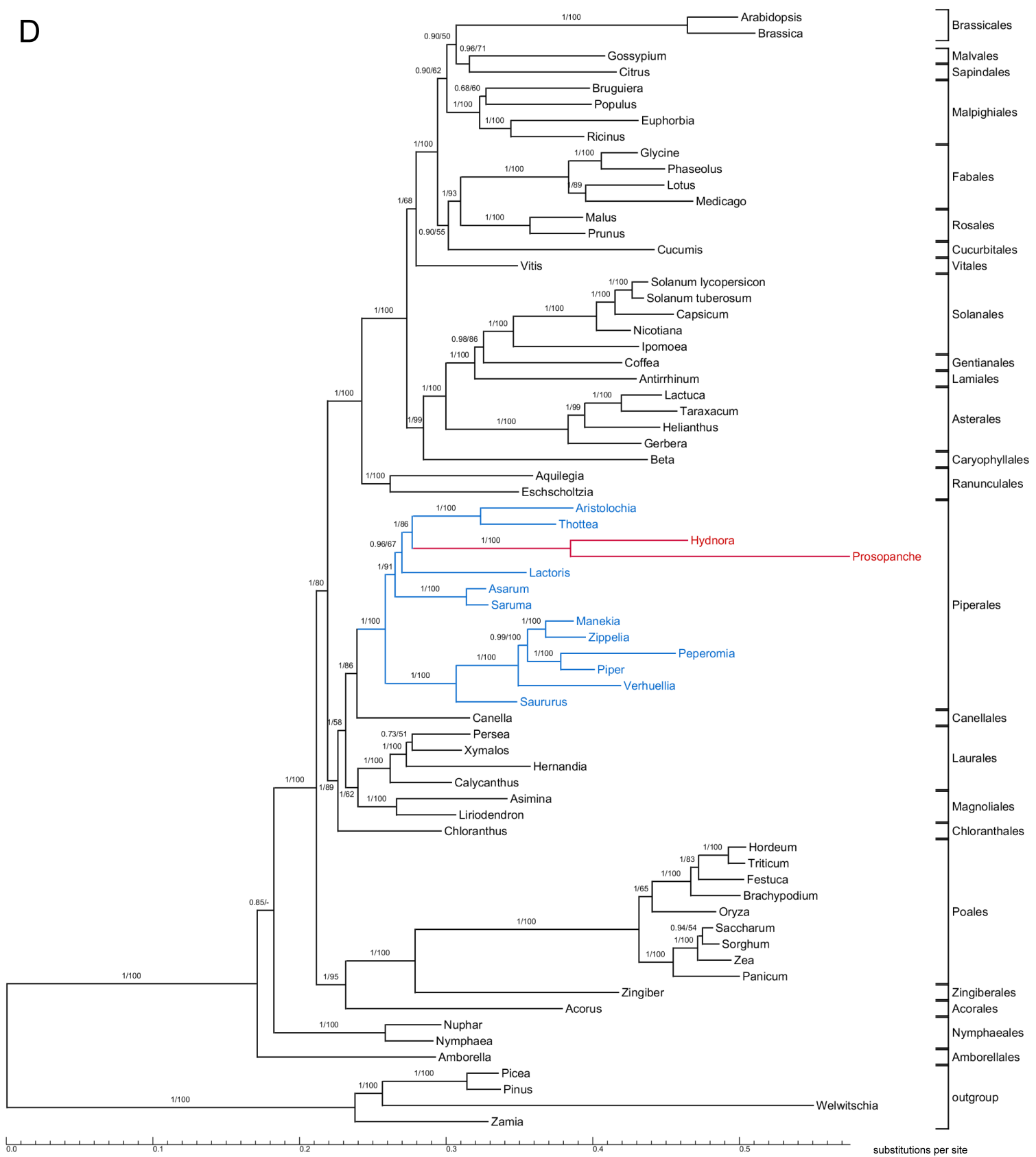

E

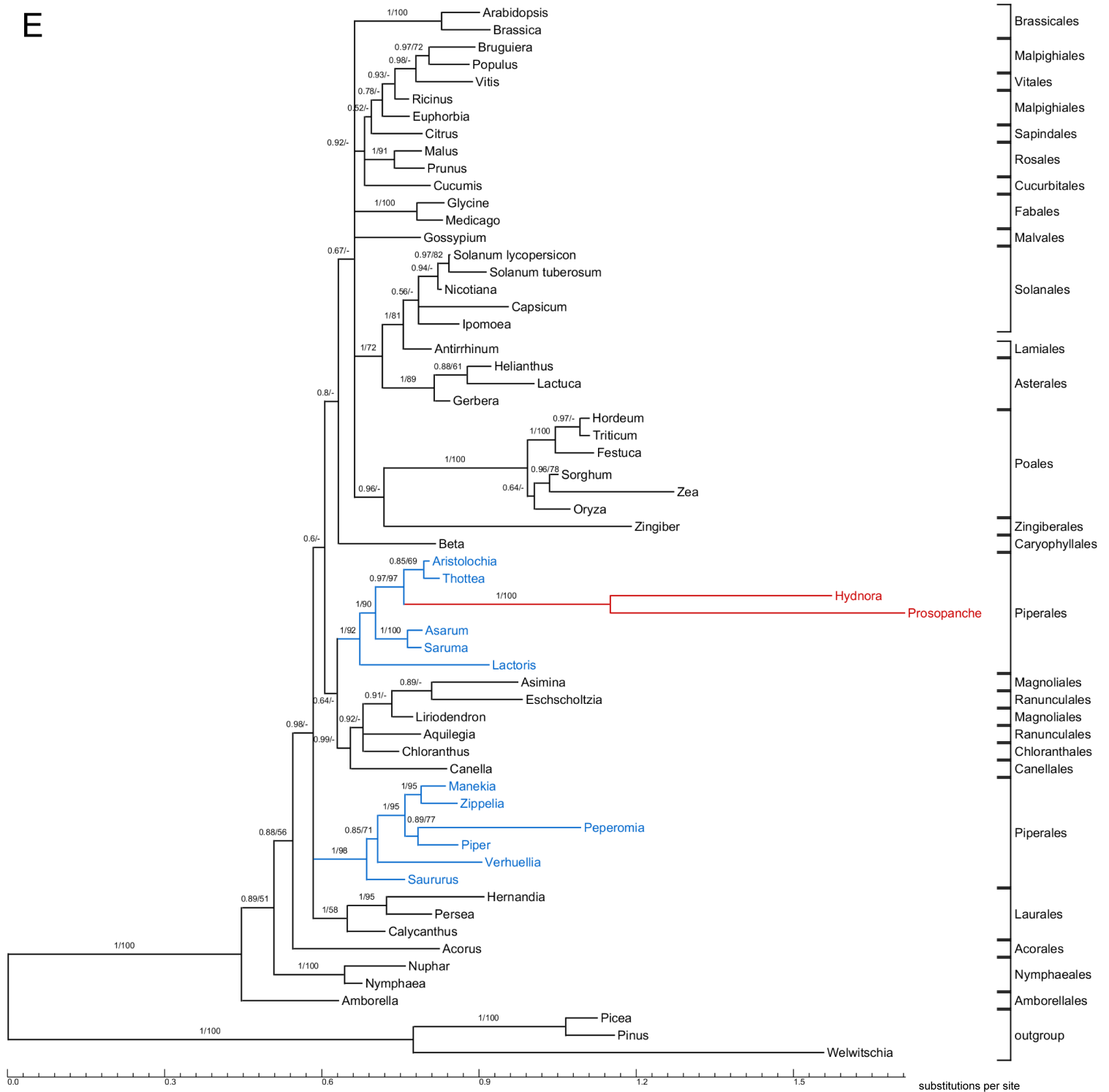

F

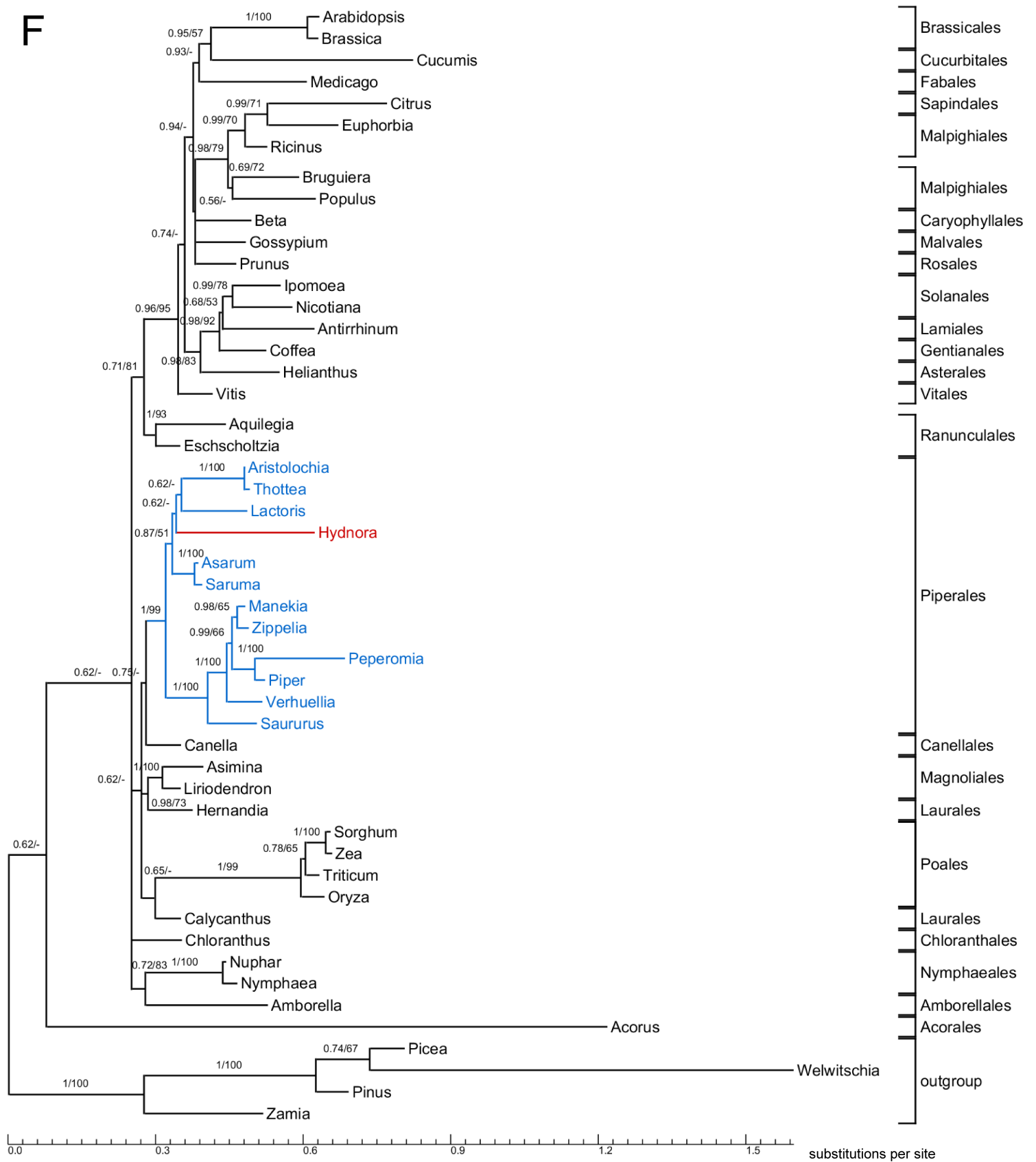

G

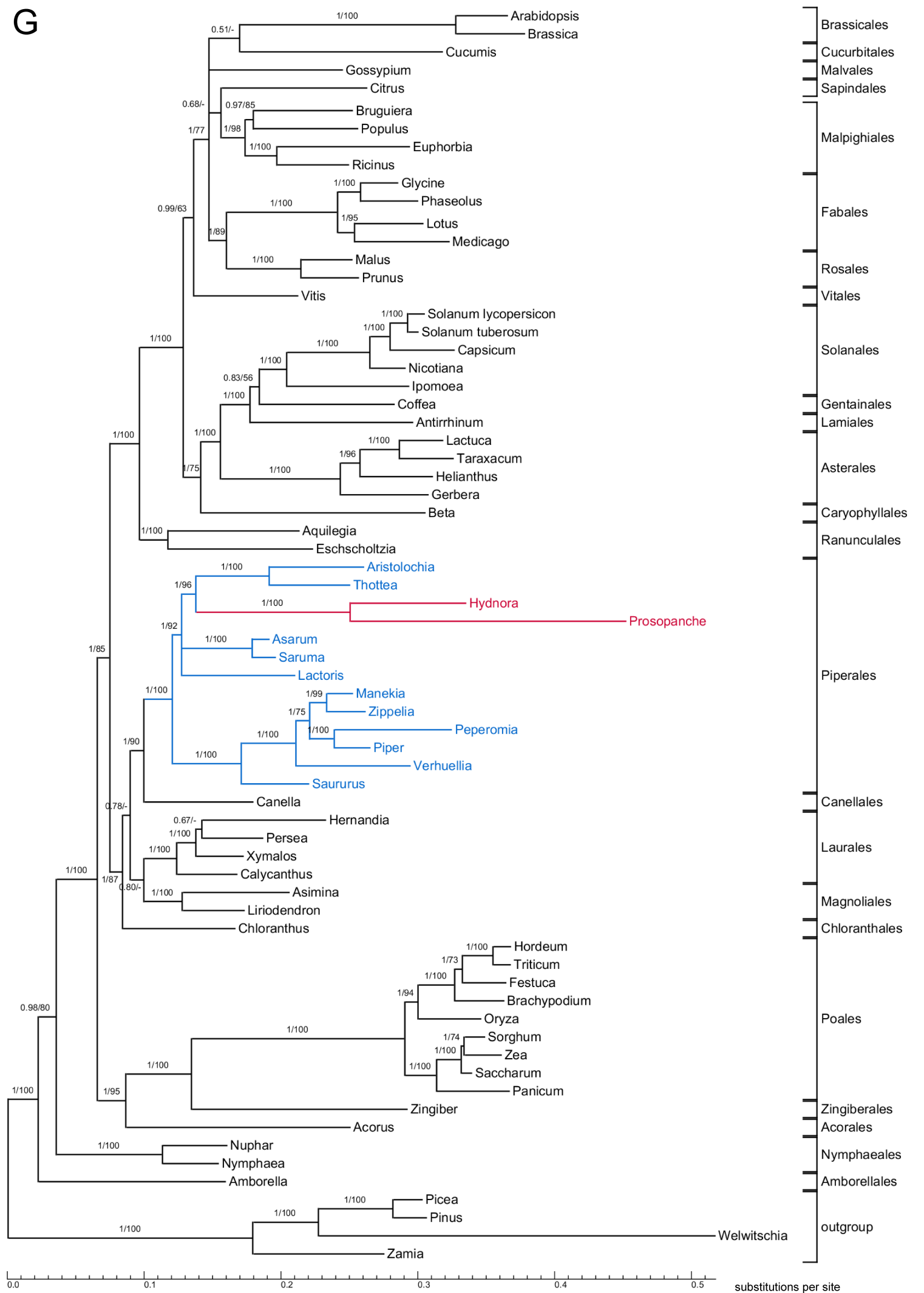

H

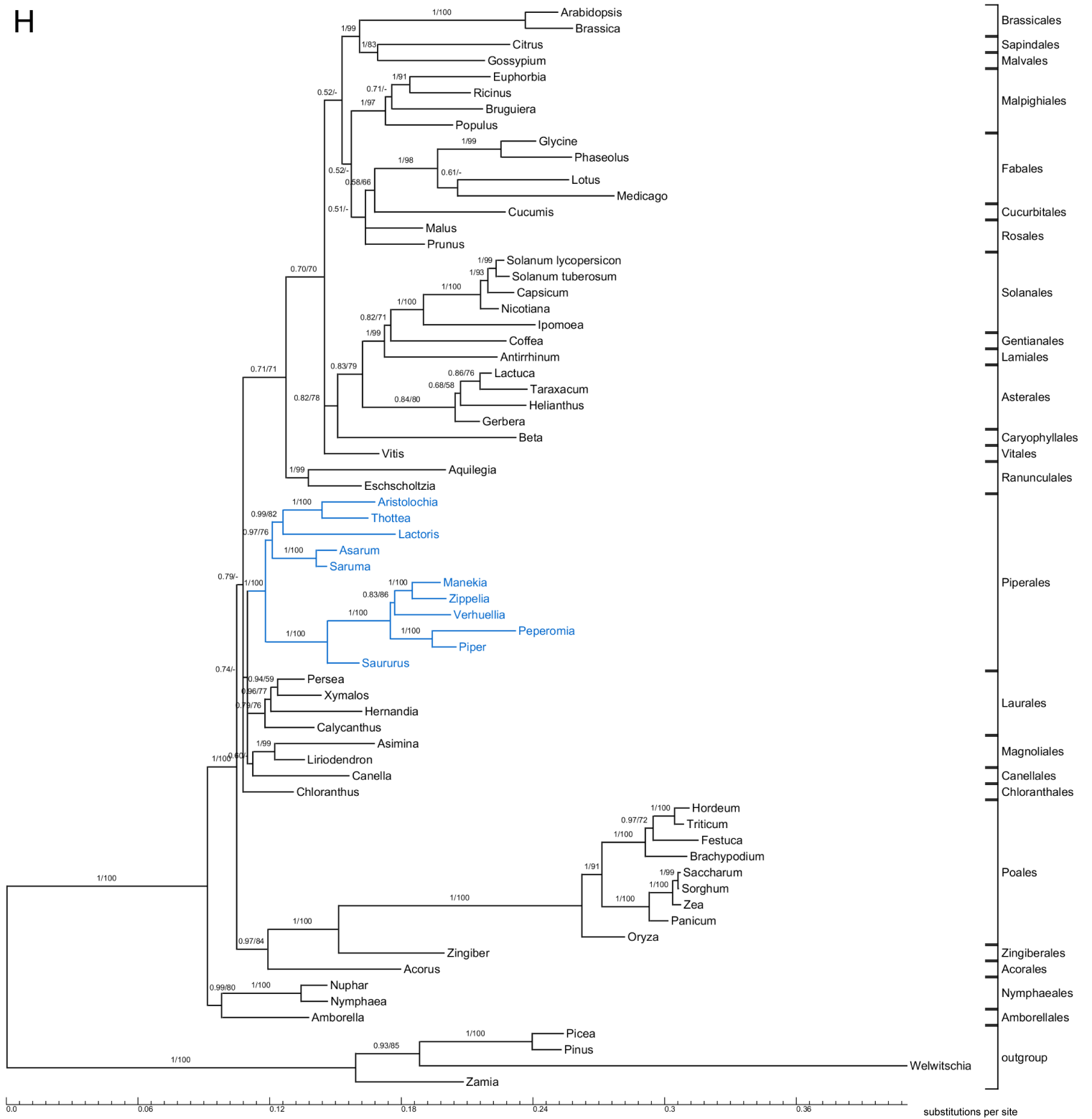

Supplement: Figure S1 — Phylogram of: A) traditional markers (18S, rbcL, atpB, atpA, and matR), B) nuclear markers (nSCG and 18S), C) nSCG only, D) 19-gene-matrix (nSCG, rbcL, atpB, atpA, and matR), E) nuclear ribosomal marker (18S) only, F) mitochondrial marker (atpA and matR) only, G) nSCG, nuclear ribosomal marker (18S), and mitochondrial marker (atpA and matR), H) plastid marker (rbcL and atpB) only, obtained from BI. Support values were mapped above branches: PP on left, BS obtained from ML on right. BS values below 50% are indicated with a dash. This figure is related to Figure 2, where a summary of this phylogenetic tree is shown. (PDF) [file pone.0079204.s001.pdf]

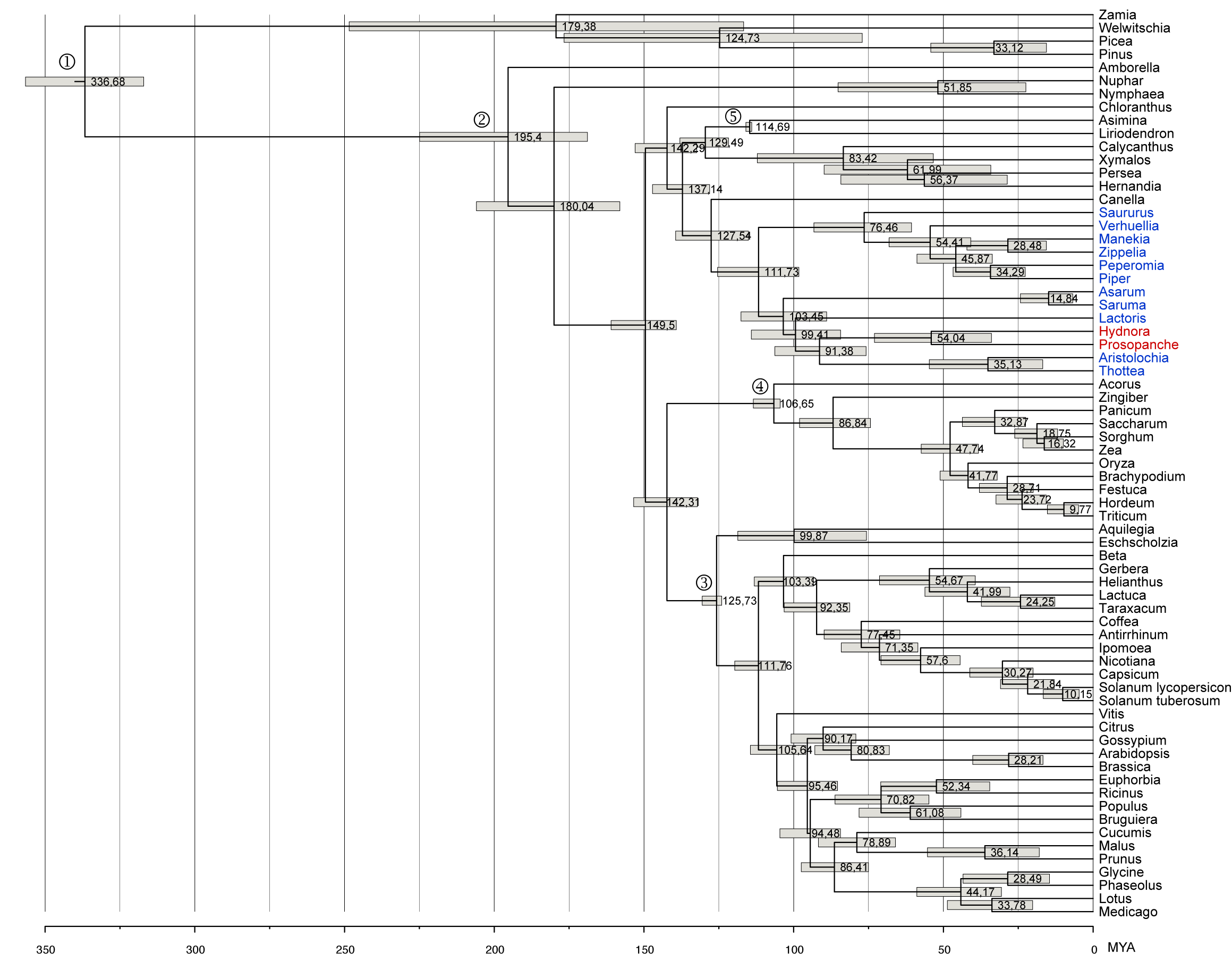

Supplement: Figure S2 — A chronogram, based on the full dataset but excluding the two chloroplast regions, applying a relaxed molecular clock using BEAST shows Hydnoraceae (red) originating in the Late Cretaceous (91 MYA) with a crown age of 54 MYA. The photosynthetic members of Piperales are highlighted in blue. The age is mapped on the right of the respective node in MYA and the highest posterior density (HPD) interval is indicated by a grey bar. Identical calibration points and topology constraints have been applied to all datasets to ensure comparability (Table 2, see methods for details). (TIF) [file pone.0079204.s002.tif]

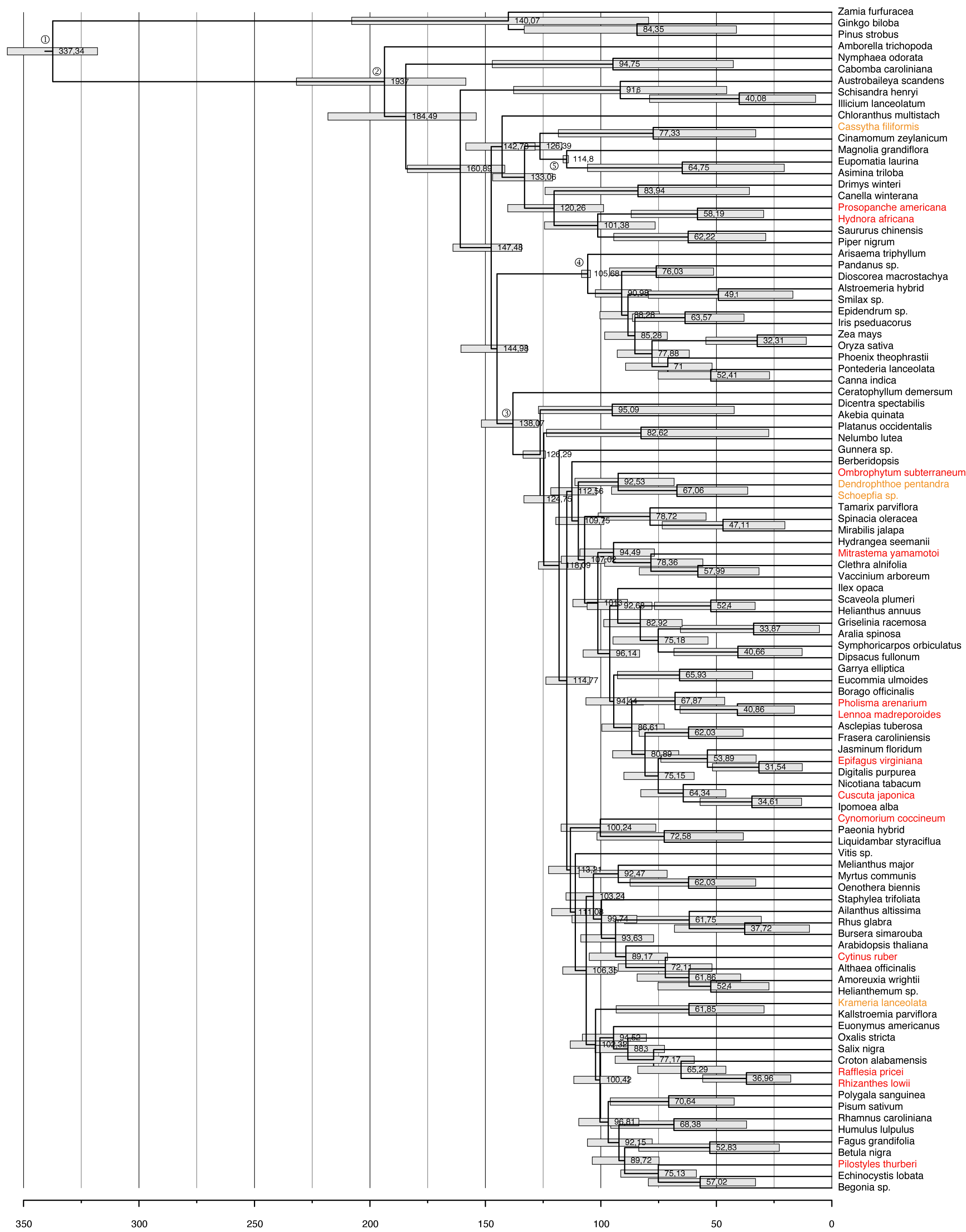

Supplement: Figure S3 — Chronogram of the mitochondrial marker dataset [10], related to Table 2. This figure shows the calculated ages obtained from the relaxed molecular clock analyses inferred in BEAST including 95% HPD intervals and applied age constraints. Identical calibration points and topology constraints have been applied to all datasets to ensure comparability (Table 2, see methods for details). (TIF) [file pone.0079204.s003.tif]
